# Prevalence of the double burden of malnutrition and its association with ideal cardiovascular health in Brazilian children

**DOI:** 10.1371/journal.pone.0349440

**Published:** 2026-06-03

**Authors:** Raytta Silva Viana, Marcus Vinicius Nascimento-Ferreira, Augusto César Ferreira De Moraes

**Affiliations:** 1 Department of Epidemiology, School of Public Health, University of São Paulo, São Paulo, São Paulo, Brazil; 2 Research Group on Health, Physical Activity and Behavior (HEALTHY-BRA), Federal University of Tocantins, Miracema do Tocantins, Tocantins, Brazil; 3 The University of Texas, Cardiovascular‑Brain Health and Lifestyle Epidemiology Laboratory (CABLES Lab), Austin, Texas, United States of America; University of Illinois Urbana-Champaign, UNITED STATES OF AMERICA

## Abstract

**Introduction:**

The Double Burden of Malnutrition (DBM), characterized by the coexistence of overweight/obesity and nutritional deficiencies, represents a growing risk to child health, affecting Ideal Cardiovascular Health (CVH). This study aimed to assess the prevalence of DBM and examine its association with CVH.

**Methods:**

The SAYCARE study was conducted in public and private schools in São Paulo and Fortaleza, including children aged 3–10 years. CVH was evaluated based on the eight components recommended by the American Heart Association. DBM was defined as the coexistence of overweight/obesity (classified by BMI according to age- and sex-specific percentiles) and iron deficiency (serum ferritin concentration <50 µg/L). Data collection included parent questionnaires, anthropometric assessments, accelerometer measurements, blood pressure, and blood samples. Descriptive statistics and multilevel Poisson regression models were applied to estimate prevalence ratios (PR) with 95% confidence intervals (95% CI), with p < 0.05 considered significant. Cluster analyses were also performed using hierarchical and non-hierarchical methods.

**Results:**

The sample included 611 children, of whom 35.96% had low CVH. DBM was identified in 4.45% of the children, characterized by the coexistence of overweight/obesity and iron deficiency. Children with overweight had a 43.06% prevalence of low CVH, while those with obesity had 49.06%. Poisson regression analysis found no significant associations between DBM and CVH; however, factors such as maternal education and family income positively influenced CVH.

**Conclusion:**

This study showed that most Brazilian children present moderate to high cardiovascular health, although a significant proportion remains at risk due to factors such as poor diet, sedentary behavior, and excess weight. The presence of DBM highlights the complexity of the nutritional transition in Brazil. Although no significant association between DBM and CVH was found, multifactorial interventions are needed to address the social, behavioral, and biomedical determinants of cardiovascular health in childhood.

## Introduction

The changes observed in recent decades in eating habits and lifestyle behaviors have resulted in the paradoxical coexistence of undernutrition and overweight within populations, families, and even individuals, a phenomenon known as the Double Burden of Malnutrition (DBM) [1,2]. In the pediatric context, DBM may be even more concerning, as it directly affects children’s growth, cognitive development, and future health, thereby compromising their physical and intellectual development potential [3,4]. The coexistence of nutritional deficiencies, such as iron deficiency anemia, and excess weight in the same child can exacerbate negative health impacts, increasing the risk of chronic diseases and compromising quality of life [5,6]. DBM therefore represents a critical barrier to achieving the Sustainable Development Goals (SDGs), particularly those related to zero hunger (SDG 2) and good health and well-being (SDG 3), which aim to ensure food security and promote healthy lives at all ages [7,8].

Although iron deficiency and overweight are widely studied as independent public health problems, the coexistence of these two conditions in the same child, a specific manifestation of DBM, remains underexplored in the scientific literature [3,9]. Few studies have investigated how these conditions may coexist and interact within an individual, especially in contexts of nutritional transition such as Brazil, where traditional undernutrition persists while childhood obesity is rapidly emerging [10,11].

Overweight and nutritional deficiencies, when present in childhood, can have significant and long-lasting impacts on cardiovascular health [12,13]. Overweight is associated with early alterations in the cardiovascular system, such as increased blood pressure, dyslipidemia, and insulin resistance, factors that predispose individuals to atherosclerosis and cardiovascular diseases (CVDs) in adulthood [14–16]. On the other hand, iron deficiency, one of the most prevalent nutritional deficiencies worldwide, not only impairs cognitive and immune development but may also lead to metabolic and cardiovascular alterations, such as reduced cardiac capacity and increased risk of hypertension, especially when occurring during critical growth periods [17–19]. The coexistence of these two conditions in the same child may further aggravate these risks, compromising cardiovascular health from early life and increasing the likelihood of chronic diseases in adulthood [6,20].

Child cardiovascular health has become an increasing concern over the past decades, as the prevalence of risk factors associated with CVDs has risen significantly among children and adolescents [21,22]. Although most CVDs are diagnosed in adulthood, robust evidence indicates that risk factors may develop early, often during childhood, and persist throughout life [23,24]. The impact of these factors on long-term cardiovascular health is substantial, since conditions such as atherosclerosis, hypertension, and heart failure have their roots in vascular and metabolic alterations that begin in childhood [14,25].

Ideal cardiovascular health, as defined by the American Heart Association (AHA) in the context of *Life’s Essential 8*, encompasses metrics such as healthy diet, regular physical activity, adequate lipid and glucose levels, blood pressure control, maintenance of healthy weight, adequate sleep, tobacco abstinence, and limited exposure to environmental pollutants [26]. These indicators are essential for preventing early cardiovascular disease and ensuring healthy aging. However, the presence of DBM, especially when combined with unfavorable socioeconomic determinants, can significantly compromise children’s ability to achieve these ideal standards of cardiovascular health [5,6,9,27].

The condition of DBM is particularly concerning in low- and middle-income countries, such as Brazil, where public policies still face significant challenges in simultaneously mitigating different forms of malnutrition, such as undernutrition and obesity [9,28]. This scenario contributes to the multiplication of cardiovascular risk factors among children, especially those in vulnerable situations, including the youngest, those from low-income families, and those exposed to humanitarian crises or food insecurity [3,29,30]. Determinants such as low maternal education, insufficient family income, and limited access to nutritious foods and health services may play a crucial role in perpetuating DBM, yet remain underexplored in epidemiological studies [30,31].

DBM, by combining nutritional deficiencies such as iron deficiency and excess weight, may be associated with a less favorable cardiovascular health profile, particularly in contexts of social inequality and rapid nutritional transition, where access to nutritious foods and health services is limited. In this context, assessing the prevalence of DBM and examining its association with a composite indicator of global cardiovascular health becomes important for the development of more effective public health policies. Such policies must be capable of addressing, in an integrated way, the various aspects of child nutrition and health, aiming to prevent chronic diseases in adulthood and contributing to reducing health inequalities and improving the quality of life of future generations.

## Materials and methods

### Study design

This study refers to the baseline analysis of the SAYCARE project, entitled *“New Perspectives on Pediatric Nutritional and Cardiovascular Health: Strategies for Assessing the Double Burden of Malnutrition and Ideal Cardiovascular Health in Low- and Middle-Income Countries – SAYCARE Cohort Study.”* It is a multicenter, cross-sectional, school-based study designed to investigate the relationship between the double burden of malnutrition (DBM) and ideal cardiovascular health (CVH) in children and adolescents [32].

### Study population and sample size

The study included preschool and school-aged children enrolled in public and private schools in São Paulo and Fortaleza, Brazil. These cities were selected for having populations exceeding 500,000 inhabitants and for hosting specialized research centers, ensuring both representativeness and feasibility. The sampling design aimed to capture the cultural and socioeconomic diversity of urban areas through a two-stage stratified random sampling process, thereby ensuring a comprehensive and balanced approach.

First stage: Schools were randomly selected based on type (public or private) and size, ensuring proportional representation across population strata defined by sex and age. The total number of eligible schools serving the target age group was first identified; eligible schools were defined as public and private schools in the selected cities with enrolled children in the target age range (3–10 years). Schools were then randomly selected in proportion to the distribution of school types.

Second stage: Within selected schools, classes were randomly sampled using stratified randomization to ensure age representativeness. The allocation was proportional, guaranteeing that the distribution of students by age group accurately reflected the target population. This methodological approach sought to maximize the precision and validity of the results.

All children enrolled in the selected classes were invited to participate. However, inclusion in the final analytical sample depended on parental or guardian consent and completion of all required assessments. Therefore, not all invited children were included in the final analysis.

The sample size was calculated considering a significance level of 5% (α = 0.05), a statistical power of 80%, and an expected prevalence of the outcome (Ideal Cardiovascular Health) of 13.9%, with a margin of ±4 percentage points. To account for the multistage sampling design, a design effect of 2.0 was applied, resulting in an initial estimate of at least 470 children. To compensate for possible losses and refusals, an additional 10% was added, followed by another 10% increase to ensure feasibility of multivariate analyses. Thus, the final minimum sample size was approximately 569 participants. This sample size not only meets statistical requirements but also aligns with the objectives of a broader health survey requiring robust samples for simultaneous evaluation of multiple outcomes.

### Ethical principles

The project was conducted in accordance with the ethical principles for research involving human subjects, as established by the Declaration of Helsinki. The SAYCARE study protocol received formal approval from the Research Ethics Committee on Human Beings of the School of Medicine, University of São Paulo (Faculdade de Medicina da Universidade de São Paulo—FMUSP), São Paulo, Brazil (CAAE: 04900918.4.1001.0065; opinion number: 3.120.232; approval date: January 24, 2019). Written informed consent was obtained from parents or legal guardians prior to participants’ enrollment. The project also complied with local regulations on human research in each participating city.

### Data collection protocol

The recruitment of participants began on September 1, 2019, and ended on December 8, 2022. Inclusion criteria comprised children aged 3–10 years whose parents or legal guardians voluntarily consented to their participation by signing the Informed Consent Form (ICF). Only children who completed all required clinical assessments, including fasting blood collection and anthropometric measurements, were included in the final analytical sample. Exclusion criteria included: parents’ inability to complete questionnaires, lack of consent from the child, and absence of fasting before blood collection.

### Materials and data collection timeline

Various measurement methods were employed, including equipment for anthropometric assessment, automated oscillometric blood pressure monitors, blood collection materials, accelerometers, and a series of questionnaires.

**Fig 1:**
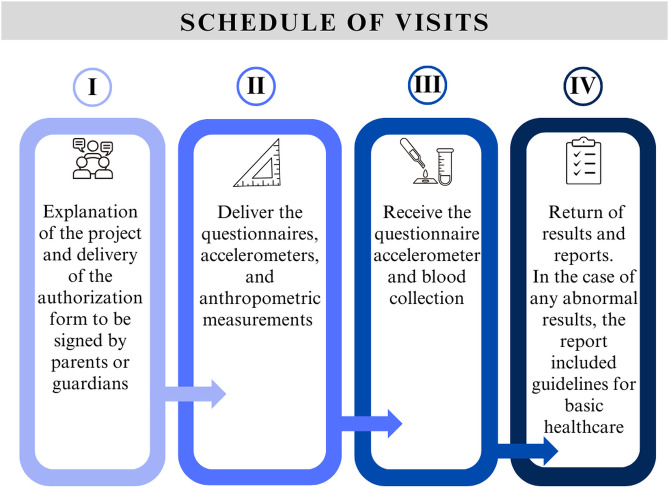
Timeline of visits and procedures for data collection in the SAYCARE study.

Data collection occurred in four main stages, distributed over sequential visits (Fig. 1):

This protocol ensured the systematic and organized collection of data, guaranteeing the quality and integrity of the information obtained.

### Variable descriptions

**Fig 2:**
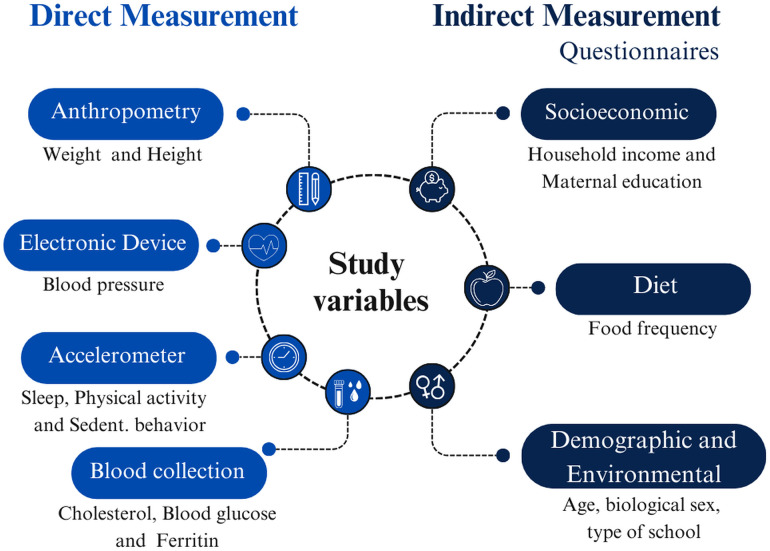
Study variables and data collection methods.

The study variables were collected through both direct and indirect measures (Fig 2).

The direct measures included: (1) Anthropometric assessments, such as weight and height, to determine nutritional status; (2) Blood pressure measurement using an electronic oscillometric device; (3) Monitoring of physical activity, sleep, and sedentary behavior using accelerometers; and (4) Blood collection for the analysis of biochemical markers such as glucose, cholesterol, ferritin, C-reactive protein (CRP), and other metabolic parameters.

The indirect measures were obtained through questionnaires, covering: (1) Environmental and demographic factors, such as biological sex, type of school, and age; (2) Socioeconomic factors, including family income and maternal education; and (3) Dietary habits, assessed through a food frequency questionnaire.

This multidimensional approach allows for a comprehensive analysis of the factors associated with children’s health and development.

### Operational definition of variables

The previously described variables were operationalized for inferential analyses as follows: biological sex (female/male), age group (3–5 and 6–10 years), DBM, and CVH were treated as categorical variables. Age was included in the models as a dichotomous variable. DBM was modeled as a binary variable (presence versus absence). CVH was dichotomized into Low (0–49 points) and Moderate/High (50–100 points) and used as the outcome in mixed-effects Poisson regression models to estimate prevalence ratios.

### Anthropometry

Anthropometric measurements were performed according to the WHO Anthropometric Standardization Manual [33]. Body weight was measured using a calibrated digital scale (Omron® HN-289, Omron Healthcare Co., Kyoto, Japan) with a flat platform (precision ± 0.1 kg), and height was measured using a portable stadiometer (SECA 213, SECA GmbH & Co. KG, Hamburg, Germany) with a vertical rule and sliding headpiece (precision ± 0.1 cm).

Each anthropometric variable was recorded once, following this order, and then repeated once or twice, maintaining the same sequence. A third measurement was performed only if the difference between any pair of readings exceeded the maximum allowable variation for each variable (weight: 100 g; height/length: 7 mm). All instruments were calibrated according to the manufacturer’s recommendations prior to data collection. All assessments were performed by the same trained researcher at each site, in a private room at the school. Measurements were taken with minimal clothing and no shoes [34].

### Accelerometer

Physical activity level, sedentary behavior, and sleep time were assessed exclusively through objective measurement using the Actigraph MTI accelerometer (model GT9X, Manufacturing Technology Inc., Fort Walton Beach, FL, USA) [35]. Parents or guardians, as well as the children, were instructed to ensure continuous use of the device day and night for 7 consecutive days, starting the day after receiving the monitor. The accelerometers were returned 8 days later. The device was worn on the waist, attached by an elastic belt. At the time of delivery, the accelerometer was positioned on the child by a trained researcher, and parents or guardians were instructed to supervise its continuous use throughout the monitoring period.

Participants received a daily log with detailed instructions and were asked to record any removal or replacement of the device, along with the activities performed and their corresponding times. Non-wear time was defined as periods of ≥20 consecutive minutes of zero counts per minute (CPM). A valid day was defined as at least 8 hours of wear time. A valid week was defined as at least 4 valid days, including at least 1 weekend day.

Periods with 0 counts per minute (CPM) for more than 20 minutes or more than 20,000 CPM were excluded. The accelerometers were configured at 30 Hz, with an epoch length of 5 seconds. Accelerometer data collection and processing followed the SAYCARE study protocol, based on previously published methodological recommendations for pediatric accelerometry [36,37].

### Physical activity

Data analysis included overall physical activity (CPM/day) and mean daily time spent in light, moderate, and vigorous physical activity, using pediatric ActiGraph cut points derived from the Evenson calibration approach, which has been widely applied in children: (1) Light: 26–573; (2) Moderate: 574–1002; and (3) Vigorous: ≥ 1003. Moderate-to-vigorous physical activity (MVPA) was calculated as the sum of moderate and vigorous minutes per day. MVPA was dichotomized as <420 minutes/week or ≥420 minutes/week, corresponding to the recommendation of at least 60 minutes/day of moderate-to-vigorous physical activity [38,39].

### Sedentary behavior

Sedentary time was estimated as the accumulated period below 100 CPM during accelerometer wear time. For each valid day, total sedentary time (SB) was expressed in minutes/day. Since wear time varied among participants, sedentary behavior was standardized by adjusting for wear time and recalculated to a 12-hour day (720 minutes) using the formula: SB time/ total wear time × 720 minutes, resulting in total SB in minutes per day. Total sedentary time was calculated as: [(mean SB on weekdays × 5) + (mean SB on weekends × 2)]/ 7.

Participants were classified using an operational threshold of <120 minutes/day (2 hours/day) of sedentary behavior, based on pediatric sedentary behavior recommendations commonly used in the literature [40,41]. Because this reference is most often applied to recreational screen-based sedentary behavior rather than total accelerometer-derived sedentary time, this categorization was interpreted cautiously.

### Sleep duration

Sleep duration was estimated from accelerometer data during the nocturnal period between 10:00 p.m. and 6:00 a.m. Sleep time was defined as continuous periods of nighttime inactivity within this interval, excluding periods of intense movement. Mean total sleep time per night was calculated in hours/night [42,43]. For analytical purposes, participants were classified according to the operational sleep categories adopted in this study: 8–10 hours/night and <8 or >10 hours/night.

### Blood pressure

Blood pressure (BP) measurements were conducted following AHA guidelines [44]. Systolic (SBP) and diastolic (DBP) pressures were measured twice, with a two-minute interval between readings. If the second measurement differed by more than 5% from the first, a third measurement was taken.

Three cuff sizes were used for children: small (12–21 cm), medium (22–32 cm), and large (33–42 cm). The appropriate cuff size was essential for accuracy, as a cuff that is too large or too small relative to the arm circumference can overestimate or underestimate BP values.

Measurements were performed on the right arm, with the arm supported at heart level, in a quiet room. Children were seated, with their back supported, feet on the floor, and arm resting on a firm surface. After 5 minutes of rest, the measurement began.

The device used was the Omron HEM-7200, a validated oscillometric digital BP monitor with an automatic inflation/deflation mechanism and a measurement range of 0–299 mmHg. This model has been validated for children and adolescents. The device was calibrated every 30 days to ensure accuracy [45,46].

### Blood sample collection procedures

Blood samples were collected using a Vacutainer system (Becton Dickinson, UK) [47]. Collection was performed in the early morning, after a 10–12-hour overnight fast, pre-scheduled with the school. Parents were instructed to maintain fasting and informed that breakfast would be provided after collection.

Qualified nurses collected 20 mL of venous blood from the antecubital vein. These were distributed into: (1) 5 mL EDTA tube (1 mg/mL); (2) 5 mL heparinized tube; and (3) 5 mL serum tube.

The protocol included: (1) Samples rested for 10 minutes at room temperature; (2) Centrifugation for 15 minutes at 3,000 rpm; and (3) Storage at –70°C.

For hemoglobin, hematocrit, and other hematological analyses, a heparinized tube was added, processed within 2 hours after collection. Samples were transported under optimal conditions to a specialized laboratory, where hematologic and metabolic analyses were performed.

### Questionnaires

All questionnaires were derived from the SAYCARE Study, previously developed and tested for reliability. For children aged 3–10 years, parents or guardians completed the questionnaires [32].

#### Demographic factors questionnaire.

Included questions about sex and age (date of birth).

#### Socioeconomic factors questionnaire.

This included 14 questions regarding household assets, domestic services, family income, parents’ education level, and current employment status [48].

Family Income: Monthly income was categorized into five groups based on the national minimum wage at data collection: *Low Income, Lower-Middle Income, Middle Income, Upper-Middle Income, and High Income*, following World Bank classifications [49].

Maternal Education: Classified into four levels — *Elementary School, High School, Technical Education,* and *University Degree* — according to WHO (2010) recommendations for studies on social determinants of health [50].

Family income was retained as the primary household socioeconomic indicator because it was directly reported and was interpreted jointly with maternal education, capturing complementary dimensions of socioeconomic position.

Food Frequency Questionnaire (FFQ) — Dietary intake was assessed through a validated FFQ [51], divided into 11 food groups: (1) Cereals and baked goods, (2) Tubers, (3) Vegetables, (4) Fruits, (5) Oils and olive oil, (6) Meats, fish, and eggs, (7) Milk and dairy products, (8) Legumes, (9) Beverages, (10) Sweets and snacks, (11) Sauces.

Parents or guardians indicated consumption frequency using a 9-point scale.

For the diet metric, dietary variables from the SAYCARE FFQ were operationalized according to the American Heart Association Life’s Essential 8 dietary framework, using the food items available in the questionnaire. Because the SAYCARE FFQ did not exactly reproduce all items of the original framework, the diet metric was adapted to the available dietary variables while preserving the component-based logic of the LE8 construct. FFQ responses were recoded into binary dietary components (criterion met vs. not met) using age-group–specific frequency thresholds.

The following components were derived: olive oil, fruits (fruit plus natural juice), butter/creams, fast food, sweets/bakery products and sugar-sweetened beverages, fish, red/processed meats, whole grains, vegetables, poultry, cheeses, and legumes/beans. These components were summed to generate a composite diet score, which was then converted into LE8 diet points according to age-group–specific operational scoring rules used in the study.

### Main variable: Double burden of malnutrition (DBM)

DBM was defined at the individual level, focusing on the coexistence of childhood overweight/obesity and iron deficiency.

Childhood overweight and obesity were classified according to the International Obesity Task Force (IOTF) age- and sex-specific BMI cutoffs proposed by Cole et al. [52]. These cutoffs are based on internationally derived reference curves and correspond to adult BMI values of 18.5 kg/m² (thinness), 25 kg/m² (overweight), and 30 kg/m² (obesity) at 18 years of age.

Iron Deficiency: Defined as serum ferritin <50 ng/mL, according to Namaste et al. (2017) and WHO recommendations [53].

DBM was characterized by the coexistence of overweight/obesity and iron deficiency in the same child.

### Outcome variable: Ideal cardiovascular health (CVH)

CVH was defined using the American Heart Association’s “Life’s Essential 8” framework [26], which includes 8 metrics divided into two domains:

Domain 1 – Health Behaviors: (1) Diet – Frequency of consumption across 12 food groups, scored 0–100 points; (2) Sleep – “Sleep – Nighttime sleep duration, classified according to the age-specific thresholds adopted for the cardiovascular health metric in this study; and (3) Physical Activity – 100 points for ≥420 min/week of MVPA, consistent with the pediatric recommendation adopted in the AHA framework.

Domain 2 – Health Factors: (1) BMI – 100 for normal, 70 for overweight, 30 for obese; (2) Non-HDL Cholesterol – 100 for <100 mg/dL, 0 for ≥190 mg/dL; (3) Glucose – 100 for <100 mg/dL, 50 for 101–125 mg/dL, 0 for ≥126 mg/dL; and (4) Blood Pressure – classified using pediatric systolic and diastolic blood pressure percentiles specific for age, sex, and height, with categorization based on cutoffs corresponding to the 50th, 90th, 95th, and 99th percentiles for the operational definition of the CVH score.

Since participants were children, smoking was excluded. The global CVH score was calculated as the mean of the 7 remaining metrics and classified as follows: (1) Moderate/High CVH: 50–100 points and (2) Low CVH: 0–49 points. Age-specific pediatric thresholds recommended by the AHA Life’s Essential 8 framework were applied whenever applicable, particularly for sleep duration, physical activity, and blood pressure.

**Fig 3:**
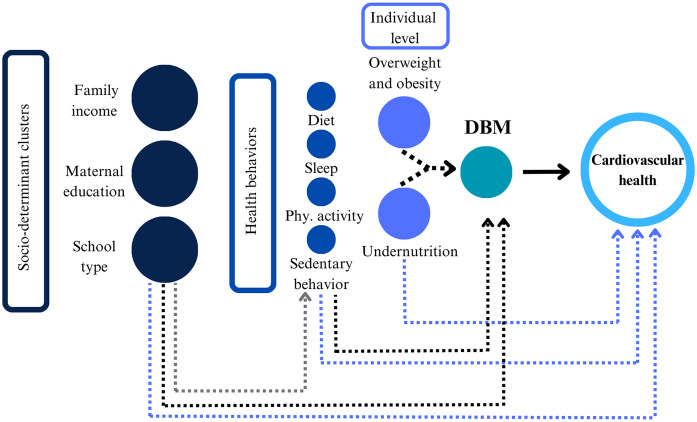
Socioeconomic clusters, health behaviors, and cardiovascular health in the double burden of malnutrition.

In this study, CVH was operationalized as a composite indicator of overall cardiovascular health status based on the AHA Life’s Essential 8 framework, rather than as a downstream causal outcome of each individual metric included in the score. Therefore, analyses involving DBM and CVH should be interpreted as examining the association between DBM and a summary cardiovascular health construct (Fig 3).

### Statistical analysis

All statistical analyses were conducted using Stata version 15.0 (Stata Corporation, College Station, TX, USA). Various statistical methods were applied, and for all hypothesis tests, the criterion for statistical significance was set at α = 0.05.

### Descriptive analyses

Descriptive analyses included the calculation of means, standard deviations, percentages, and 95% confidence intervals, stratified by city. These analyses provided an overview of the demographic and behavioral characteristics of the study participants.

Normality of continuous variables was assessed using graphical inspection and appropriate normality tests when applicable.

### Exploratory socioeconomic cluster analysis

Exploratory cluster analysis was conducted as a complementary descriptive approach to summarize the joint distribution of key socioeconomic indicators in the sample [54]. Three social variables were considered: family income, type of school (public or private), and maternal education (Incomplete Basic Education; Completed Elementary School; Completed High School; Completed Higher Education). Family income was included because, together with maternal education and school type, it captures a complementary dimension of household socioeconomic position and helps characterize combined socioeconomic profiles rather than isolated indicators

Three groups were identified, each representing a distinct socioeconomic profile derived from the combination of these indicators. These clusters were used for descriptive characterization only and were not intended to replace the conventional socioeconomic variables included separately in the regression models:

Cluster 1 – BEBR: combines low maternal education and low family income;
Cluster 2 – EMVE: represents medium maternal education and family economic vulnerability;
Cluster 3 – AEMC: includes high maternal education and higher socioeconomic status.

A strategy combining hierarchical and non-hierarchical clustering was adopted to identify groups with similar socioeconomic profiles. Initially, complete linkage hierarchical clustering was applied based on squared Euclidean distances. The initial solution was then refined using non-hierarchical k-means clustering, using the resulting centroids. This procedure was used to support an exploratory descriptive grouping of children according to combined socioeconomic characteristics.

**Fig 4:**
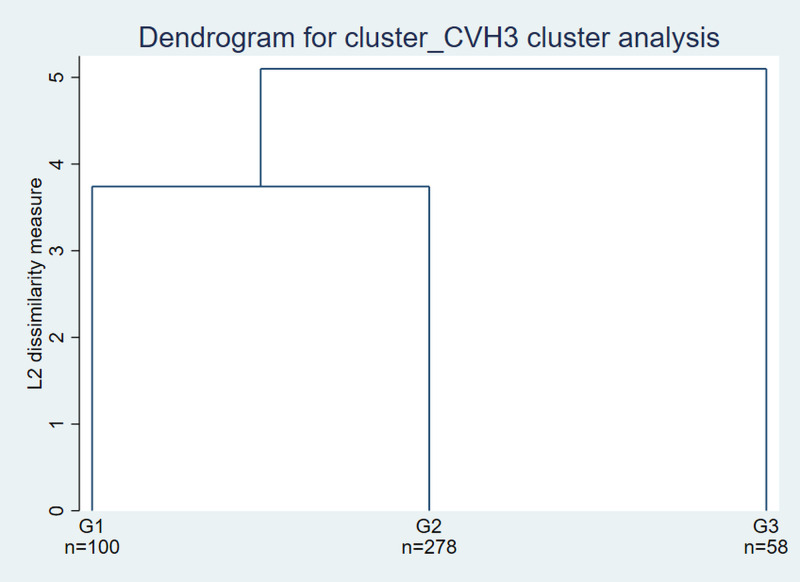
Dendrogram showing the distribution of identified clusters.

To visualize the hierarchical structure of the clusters, a dendrogram was created, showing how observations were grouped as clusters were combined (Fig 4). This visualization helped determine the appropriate number of clusters for the study, allowing categorization of children into groups with different cardiovascular risk profiles based on specific social and economic determinants.

### Multilevel analyses

Multilevel Poisson regression models with random intercepts were used to estimate prevalence ratio (PR) coefficients and their 95% confidence intervals for variables of interest and potential confounders. In the multilevel regressions, the contextual variable considered was the school and/or city. The association models examining DBM in relation to CVH were treated as exploratory and interpreted with caution because DBM includes overweight/obesity, while BMI is also a component of the composite CVH score.

The association between ideal cardiovascular health and several covariates was analyzed, considering two levels of data organization: (1) Contextual factors: city (research center), maternal education, family income, and type of school; and (2) Individual factors: sex, age, physical activity, sedentary behavior, sleep time, and DBM.

A 10% change in the β coefficient of any variable already present in the model was considered significant. Variables with p < 0.20 in univariate analysis were included in the multivariable multilevel models. The level of statistical significance was set at p < 0.05.

### Directed acyclic graph (DAG)

A directed acyclic graph (DAG) was developed using Dagitty (www.dagitty.net) to depict the conceptual structure underlying the multilevel analyses and to improve transparency regarding covariate inclusion. The DAG summarizes the assumed relationships among double burden of malnutrition (DBM), contextual socioeconomic indicators (family income, maternal education, and type of school), demographic variables (age and sex), city (research center), and accelerometer-derived behaviors (physical activity, sedentary behavior, and sleep duration) in relation to ideal cardiovascular health (CVH). The DAG was included as a supplementary figure (S1 Fig).

## Results

A total of 674 children initially agreed to participate in the study. However, some participants were absent on the days of clinical assessments or did not complete blood collection. Additionally, a small number of cases presented incomplete information regarding biological sex or date of birth, which precluded their inclusion in the analyses. After these exclusions, the final analytical sample consisted of 611 children.

Table 1 presents the distribution of sociodemographic, behavioral, and health characteristics of the participating children. The sample included children from two Brazilian cities (77.74% from São Paulo and 22.26% from Fortaleza), with a balanced distribution between biological sexes (52.29% male and 47.71% female). Most children attended public schools and came from low-income families (51.93% classified as low income). Maternal education varied, with the majority of mothers having completed high school (48.23%).

**Table 1:** Distribution of Independent Variables and Prevalence of Ideal Cardiovascular Health among Brazilian children in the SAYCARE Study, Brazil, 2024.

Independent Variables	Total n = 611*	Prevalence (%)	(95% CI)
**Research Center**			
Fortaleza	136	22.26%	19.12–25.74
São Paulo	475	77.74%	74.25–80.87
**School Type**			
Public	503	82.32%	79.08–85.15
Private	108	17.68%	14.84–20.91
**Maternal Education**			
Elementary School	85	17.75%	14.56–21.44
High School	231	48.23%	43.76–52.71
Technical School	40	8.35%	6.17–11.19
University Education	123	25.68%	21.95–29.79
**Household Income**			
Low	229	51.93%	47.24–56.57
Low-Medium	147	33.33%	29.07–37.88
Medium	46	10.43%	7.89–13.66
Medium-High	18	4.08%	2.58–6.39
High	1	0.23%	0.03–1.60
**Biological Sex**			
Female	271	47.71%	43.61–51.83
Male	297	52.29%	48.16–56.38
**Age Group**			
3–5 years	151	26.73%	23.22–30.54
6–10 years	414	73.27%	69.45–76.77
**Diet**			
Inadequate	337	69.77%	65.51–73.71
Moderate	142	29.40%	25.49–33.63
Ideal	4	0.83%	0.31–2.19
**Sleep** ^**a**^			
< 8 or >10 h/night	175	60.34%	54.56–65.84
8–10 h/night	115	39.66%	34.15–45.43
**Physical Activity** ^**b**^			
< 420 min/week	40	11.46%	8.50–15.27
≥ 420 min/week	309	88.54%	84.72–91.49
**Sedentary Behavior** ^**c**^			
> 2h/day	317	90.83%	87.30–93.45
≤ 2h/day	32	9.17%	6.54–12.69
**Nutritional Status**			
Underweight	29	5.34%	3.73–7.58
Ideal Weight	323	59.48%	55.28–63.55
Overweight	104	19.15%	16.04–22.69
Obesity	87	16.02%	13.16–19.36
**Blood Pressure** ^**d**^			
Hypertension	103	30.11%	25.59–35.00
Elevated Blood Pressure	194	56.73%	51.39–61.90
Ideal Blood Pressure	45	13.16%	9.95–17.19
**Cholesterol** ^**e**^			
< 120 mg/dL	350	74.31%	70.15–78.06
120–144 mg/dL	102	21.66%	18.15–25.62
≥ 145 mg/dL	19	4.03%	2.58–6.24
**Glycemia** ^**f**^			
< 100 mg/dL	470	99.79%	98.49–99.97
101–125 mg/dL	1	0.21%	0.02–1.50
**Iron Deficiency** ^**g**^			
≥ 50 ng/dL	415	87.92%	84.64–90.57
< 50 ng/dL	57	12.08%	9.42–15.35
**DBM** ^**h**^			
Without DBM	451	95.55%	93.26–97.08
With DBM	21	4.45%	2.91–6.73
**Ideal Cardiovascular Health**			
Low	123	35.96%	31.02–41.21
Moderate/High	219	64.04%	58.78–68.97

a – hours of sleep per night; b – minutes of Moderate to Vigorous Physical Activity per week; c – hours of sedentary behavior per day; d – blood pressure categories were defined using pediatric systolic and diastolic blood pressure percentiles (age-, sex-, and height-specific), with classification into ideal, elevated, and hypertensive levels according to the study’s operational scoring criteria. e – milligrams per deciliter of Non-High-Density Lipoprotein Cholesterol; f – milligrams per deciliter of glucose; g – nanograms per milliliter of ferritin; h – DBM: category of Double Burden of Malnutrition (Overweight + Iron Deficiency). *The total analytical sample comprised 611 children; however, the number of valid observations varied across variables because some measures were missing for specific assessments. Therefore, category totals may not sum to 611 in all variables.

Regarding metabolic and behavioral health, a high prevalence of unhealthy habits was observed. Inadequate diet was reported in 69.77% of the sample, 60.34% presented non-recommended sleep duration and 90.83% exceeded 2 hours/day of accelerometer-derived sedentary time according to the operational classification used in this study. Despite this, most children met the physical activity recommendations (88.54% accumulated ≥420 minutes/week of MVPA).

Nutritional status revealed that 35.17% of the children presented excess weight (19.15% overweight and 16.02% obesity), while 5.34% were underweight. Regarding blood pressure, 30.11% had hypertension and 56.73% had elevated blood pressure, totaling 86.84% with non-ideal blood pressure levels.

Concerning metabolic markers, cholesterol and glucose levels were mostly adequate (74.31% had non-HDL cholesterol <120 mg/dL and 99.79% had glucose <100 mg/dL), but 12.08% of the children exhibited iron deficiency. The presence of DBM (overweight/obesity + iron deficiency) was identified in 4.45% (n = 21) of the sample.

As for cardiovascular health, 64.04% of the children were classified as having Moderate/High CVH, whereas 35.96% were classified as Low CVH, indicating that more than one-third of the sample did not meet the criteria for ideal cardiovascular health.

Descriptive analysis showed variation in CVH prevalence across the socioeconomic clusters (Table 2). Children in the AEMC cluster presented a higher prevalence of Moderate/High CVH (75.26%) than those in the EMVE cluster (65.60%), while the BEBR cluster showed a similar prevalence (73.17%). Regional differences were also observed, with Moderate/High CVH more frequent in Fortaleza (67.69%) than in São Paulo (52.43%).

**Table 2:** Prevalence of Ideal Cardiovascular Health according to Independent Variables among Brazilian children in the SAYCARE Study, Brazil, 2024.

Independent Variable	Low CVH (95% CI)	Mod./High CVH (95% CI)
**Cluster** ^**a**^		
BEBR^(1)^	26.82% (15.36–42.54)	73.17% (57.45–84.63)
EMVE^(2)^	34.40% (26.54–43.21)	65.60% (56.78–73.45)
AEMC^(3)^	24.73% (16.95–34.58)	75.26% (65.41–83.04)
**Research Center**		
Fortaleza	32.30% (26.87–38.26)	67.69% (61.73–73.12)
São Paulo	47.56% (36.92–58.41)	52.43% (41.58–63.07)
**School Type**		
Public	33.20% (27.70–39.19)	66.79% (60.80–72.29)
Private	44.57% (39.19–55.46)	55.42% (44.53–65.80)
**Maternal Education**		
Elementary School	23.07% (13.49–36.59)	76.92% (63.40–86.50)
High School	31.61% (24.31–39.96)	68.38% (60.03–75.68)
Technical School	26.08% (11.95–47.85)	73.91% (52.14–88.04)
University Education	29.11% (20.09–40.14)	70.88% (59.85–79.90)
**Household Income**		
Low	36.50% (28.51–45.32)	63.49% (54.67–71.48)
Low-Medium	23.25% (15.45–33.43)	76.74% (66.56–84.54)
Medium	27.27% (14.64–45.03)	72.72% (59.96–85.35)
Medium-High	18.75% (5.90–45.91)	81.25% (54.08–94.09)
High	(no observations)	(no observations)
**Biological Sex**		
Female	36.20% (29.36–43.65)	63.79% (56.34–70.63)
Male	35.71% (28.79–43.28)	64.28% (56.71–71.20)
**Age Group**		
3–5 years	49.15% (36.57–61.83)	50.84% (38.16–63.42)
6–10 years	33.21% (27.94–38.94)	66.78% (61.05–72.05)
**Diet**		
Inadequate	30.76% (2483–37.41)	69.23% (62.58–75.16)
Moderate	18.82% (11.80–28.65)	81.17% (71.34–88.19)
Ideal	(no observations)	(no observations)
**Sleep** ^**a**^		
< 8 or >10 h/night	15.00% (9.19–23.51)	85.00% (76.48–90.80)
8–10 h/night	20.45% (13.21–30.28)	79.54% (69.71–86.78)
**Physical Activity** ^**b**^		
< 420 min/week	29.41% (12.38–55.12)	70.58% (44.87–87.61)
≥ 420 min/week	23.58% (18.31–29.81)	76.41% (70.18–81.68)
**Sedentary Behavior** ^**c**^		
> 2h/day	22.97% (17.87–29.01)	77.02% (70.98–82.12)
≤ 2h/day	57.14% (20.78–87.13)	42.85% (12.86–87.13)
**Nutritional Status**		
Underweight	53.33% (28.49–76.62)	46.66% (23.37–71.50)
Ideal Weight	35.35% (22.41–34.99)	71.71% (65.00–77.58)
Overweight	43.05% (32.05–54.78)	56.94% (45.21–67.94)
Obesity	49.05% (35.81–62.43)	50.94% (37.56–64.18)
**Blood Pressure**		
Hypertension	43.44% (35.00–52.07)	56.55% (47.93–65.00)
Elevated Blood Pressure	36.08% (29.59–43.11)	63.91% (56.88–70.40)
Ideal Blood Pressure	20.00% (10.64–34.41)	80.00% (65.58–89.35)
**Cholesterol** ^**d**^		
< 120 mg/dL	20.97% (15.90–27.13)	79.02% (72.86–84.09)
120–144 mg/dL	29.62% (18.88–43.22)	70.37% (56.77–81.11)
≥ 145 mg/dL	72.72% (39.72–91.51)	27.27% (8.48–60.67)
**Glycemia** ^**e**^		
< 100 mg/dL	24.62% (19.80–30.17)	75.37% (69.82–80.19)
101–125 mg/dL	(no observations)	(no observations)
**Iron Deficiency** ^**f**^		
≥ 50 ng/dL	25.10% (19.98–31.02)	74.89% (68.97–80.01)
< 50 ng/dL	22.58% (10.98–40.79)	77.41% (59.20–89.01)
**DBM** ^**h**^		
Without DBM	29.07% (22.11–37.18)	70.92% (62.81–77.88)
With DBM	5.71% (1.39–20.66)	94.28% (79.33–98.60)

a – Cluster: grouped according to School Type, Maternal Education, and Household Income = (1) low maternal education and low household income; (2) medium maternal education and family economic vulnerability; (3) high maternal education and better socioeconomic condition; b – hours of sleep per night; c – minutes of physical activity per week; d – hours of sedentary behavior per day; e – milligrams per deciliter of Non-High-Density Lipoprotein Cholesterol; f – milligrams per deciliter of glucose; g – nanograms per milliliter of ferritin; h – DBM: category of Double Burden of Malnutrition (Overweight + Iron Deficiency).

Adequate physical activity (≥420 minutes/week) was associated with a higher prevalence of Moderate/High CVH (76.41% vs. 70.58% in those below recommendations), and a moderate diet was also associated with higher CVH prevalence (81.17% vs. 69.23% in inadequate diet). Conversely, children with obesity showed a higher prevalence of Low CVH (49.05%) compared to those with ideal weight (35.35%).

Children with hypertension had a notably higher prevalence of Low CVH (43.44%) compared to those with ideal blood pressure (20.00%). Elevated non-HDL cholesterol (≥145 mg/dL) was associated with a markedly higher prevalence of Low CVH (72.72%).

The univariate analysis (Table 3) did not reveal statistically significant associations between the analyzed variables and CVH (all p-values >0.05), although nutritional status (PR = 0.890; 95% CI 0.791–1.001; p = 0.053) and cholesterol (PR = 0.773; 95% CI 0.577–1.036; p = 0.085) showed borderline trends.

**Table 3:** Prevalence Ratio (PR) of Ideal Cardiovascular Health according to Independent Variables among Brazilian children in the SAYCARE Study, Brazil, 2024.

Independent Variable	PR	95% CI	*p-*value
**Cluster** ^**a**^	1,041	0,843–1,286	0,710
**School Type**	0,830	0,599–1,149	0,260
**Maternal Education**	0,989	0,870–1,124	0,864
**Household Income**	1,089	0,936–1,268	0,269
**Biological Sex**	1,011	0,775–1,317	0,937
**Age**	1,314	0,894–1,931	0,165
**Diet**	1,176	0,896–1,543	0,242
**Sleep**	0,936	0,682–1,284	0,681
**Physical Activity**	1,079	0,595–1,960	0,802
**Sedentary Behavior**	0,556	0,178–1,742	0,314
**Nutritional Status**	0,890	0,791–1,001	0,053
**Blood Pressure**	1,004	0,998–1,010	0,206
**Cholesterol**	0,773	0,577–1,036	0,085
**Glucose**	1,327	0,186–9,464	0,778
**DBM** ^**b**^	0,811	0,400–1,645	0,562

PR = Prevalence Ratio; 95% CI = 95% Confidence Interval; a – Cluster: grouped according to School Type, Maternal Education, and Household Income; b – DBM: category of Double Burden of Malnutrition (Overweight + Iron Deficiency). P-values < 0.05 were considered significant, indicated in bold with an asterisk (*).

Regarding lifestyle habits, no statistically significant associations were observed between diet, sleep, physical activity, or sedentary behavior and CVH prevalence in the univariate analysis. Blood pressure, glycemia, and DBM were also not significantly associated with CVH. These findings should be interpreted with caution, since DBM includes overweight/obesity and the composite CVH score also includes BMI, introducing partial overlap between exposure and outcome.

The final multilevel model (Table 4) did not demonstrate statistically significant associations between socioeconomic cluster (PR = 1.017; 95% CI 0.778–1.331; p = 0.901), DBM (PR = 1.051; 95% CI 0.376–2.941; p = 0.924), or behavioral variables and CVH. The random effect of the city was negligible, and the likelihood ratio test indicated no substantial improvement compared to a simple Poisson model.

**Table 4:** Prevalence ratio of ideal cardiovascular health in the final multilevel model.

Independent Variable	PR	95% CI	*p-*value
**Cluster** ^**a**^	1,017	0,778–1,331	0,901
**Biological Sex**	0,995	0,678–1,461	0,980
**Age**	1,234	0,671–2,267	0,499
**Sedentary Behavior**	1,014	0,189–5,438	0,987
**Diet**	1,011	0,680–1,503	0,958
**Sleep**	0,993	0,669–1,474	0,972
**Physical Activity**	1,008	0,401–2,536	0,986
**DBM** ^**b**^	1,051	0,376–2,941	0,924
**_cons**	0,631	0,107–3,707	0,610

PR = Prevalence Ratio; 95% CI = 95% Confidence Interval; a – Cluster: grouped according to School Type, Maternal Education, and Household Income; b – DBM: category of Double Burden of Malnutrition (Overweight + Iron Deficiency). None of the variables showed statistical significance (p < 0.05).

Despite the lack of statistical significance, a trend toward a higher prevalence of CVH was observed in older children. The analysis also did not indicate a significant relationship between DBM and CVH, suggesting that, in the context of this study, these factors do not influence the prevalence of CVH.

Furthermore, the random effect of the city was virtually negligible, indicating that variability between cities regarding CVH is not significant. The likelihood ratio (LR) test confirmed that the mixed-effects model did not show substantial improvement compared to a simple Poisson model.

## Discussion

This study aimed to evaluate the prevalence of and test associations between DBM at the individual level and CVH in children. Although the multilevel analysis did not show a significant association between DBM and CVH, the presence of this condition in a portion of the pediatric population is concerning and warrants attention. DBM reflects the complexity of the nutritional transition in Brazil, where undernutrition and obesity coexist, often within the same individual or family [34]. This phenomenon poses a particular public health challenge, as it combines the detrimental effects of both conditions, increasing the risk of metabolic and cardiovascular comorbidities [9].

### Prevalence of DBM

Sawaya et al. were pioneers in highlighting this duality at the family level, reporting that 9% of Brazilian households had members living with both overweight and undernutrition [53]. In the present study, we observed that this coexistence also manifests at the individual level, with a prevalence of 4.45% of Brazilian children simultaneously presenting obesity and iron deficiency. Our findings show a prevalence higher than the 2.3% estimate reported for Brazil in a meta-analysis evaluating DBM in Latin American children from 1988 to 2017 [55]. This meta-analysis, which included 88 estimates, found a combined prevalence of overweight and stunting of 1.6% (95% CI: 0.016–0.017) for Latin America as a whole, highlighting Brazil with a prevalence of 2.3% [54].

The higher prevalence observed in the present study may reflect methodological differences, temporal changes in the population’s nutritional profile, or specific characteristics of the analyzed sample. However, these results reinforce the complexity of the nutritional transition in Brazil, suggesting that malnutrition, whether due to excess or deficiency, does not remain confined to family dynamics but is also expressed individually, possibly reflecting socioeconomic inequalities, inadequate access to nutritious foods, and changes in dietary patterns and physical activity behaviors.

### Iron deficiency and obesity

This study found a prevalence of 12.08% of iron deficiency among the children. This result is relevant, as iron deficiency is a well-established risk factor for anemia development and for impaired cognitive and physical development in children [56]. Its importance is even greater when considering the complex relationship between iron deficiency and obesity. A systematic review and meta-analysis including 41 studies with a total of 37,500 children with obesity revealed that obesity is significantly associated with iron deficiency, with an odds ratio (OR) of 2.1 (95% CI: 1.4–3.2) [57]. This indicates that overweight children have a 110% higher chance of presenting iron deficiency compared to children without obesity.

This relationship can be explained by complex biological mechanisms, in which low-grade chronic inflammation plays a central role. Obesity is characterized by a persistent inflammatory state mediated by increased production of proinflammatory cytokines, such as interleukin-6 (IL-6) and tumor necrosis factor-alpha (TNF-α). These cytokines stimulate the production of hepcidin, a hormone that regulates iron metabolism in the liver. Hepcidin reduces intestinal iron absorption and inhibits the release of iron from macrophages and hepatic stores, resulting in decreased circulating iron availability [58].

There is a bidirectional relationship between obesity and iron deficiency: obesity promotes inflammation and increased hepcidin, leading to iron deficiency, while iron deficiency may exacerbate metabolic complications associated with obesity, such as insulin resistance and chronic inflammation. Iron deficiency can worsen mitochondrial dysfunction and oxidative stress, mechanisms that contribute to increased insulin resistance and the inflammatory profile in individuals with obesity. This interaction creates a vicious cycle in which obesity and iron deficiency reinforce each other, amplifying health risks [58].

This complex relationship may further harm children’s health, as iron deficiency can exacerbate the negative effects of excess weight. In our findings, although no direct association between DCMN and CVH was identified, 22.58% of children with low CVH presented iron deficiency. These results suggest that iron deficiency, in combination with obesity, may contribute to increased risk of cardiovascular complications [59], highlighting the importance of monitoring both factors in the pediatric population. Early identification and proper management of iron deficiency in children with obesity are essential to interrupt this cycle and prevent adverse health outcomes.

### Excess weight and cardiovascular health

Regarding nutritional status, excess weight was identified in 35.17% of children (19.15% overweight and 16.02% obese), which aligns with global trends observed in low- and middle-income countries [55]. This scenario reinforces the growing concern about the increase in childhood overweight, which, although recent, constitutes a public health problem with serious implications, such as an increased risk of NCDs.

Prevention of cardiovascular disease should begin in childhood, and the impact of overweight and obesity on pediatric cardiovascular health is significant. A recent study showed that children with obesity had a significantly higher percentage of developing hypertension (14.8%; 95% CI: 11.5–18.0), elevated total cholesterol and/or triglyceride levels (18%; 95% CI: 15.3–20.7), insulin resistance (12.3%; 95% CI: 9.8–14.8), and prediabetes (7.5%; 95% CI: 5.5–9.5) compared to children with normal weight [60]. Identification of excess weight and cardiometabolic risk factors, such as hypertension, dyslipidemia, and insulin resistance, is essential to prevent future complications.

Clinically, follow-up of children with excess weight should be accompanied by personalized interventions, such as lifestyle modification programs. Family involvement is crucial, with educational actions promoting healthy habits and reducing consumption of foods high in sugar, salt, and saturated fats. Additionally, attention to mental health should not be neglected, with early identification of disorders such as depression and anxiety, which can lead to risky behaviors like poor diet and sedentary lifestyle.

From a public policy perspective, integrated strategies promoting healthy environments are important, such as regulating food availability in schools and promoting safe public spaces for physical activity. Regulating marketing practices targeting children, especially for unhealthy foods, can play an important role in reducing excessive calorie consumption and improving diet quality. Implementation of these measures in an integrated, multisectoral manner can improve children’s cardiovascular health and reduce NCD burden in adulthood, ensuring a healthier future for upcoming generations.

### Health behaviors and cardiovascular health

Adequate physical activity and a balanced diet were more prevalent in the moderate/high CVH category compared to low physical activity and inadequate diet, although these associations were not statistically significant. These results align with the literature, highlighting the importance of healthy habits from childhood for preventing cardiovascular diseases in adulthood. A systematic review analyzing 53 studies with 26,045 children showed that lifestyle interventions combining nutrition and physical activity were effective in reducing BMI and improving cardiometabolic risk factors, such as adiposity, blood pressure, lipid levels, and glycemia [61].

Sedentary behavior, in turn, showed a trend toward higher prevalence of low CVH, present in 90.83% of children in our study. Research highlights sedentary behavior as a modifiable risk factor for cardiovascular health in children and adolescents. Reallocating 30–60 minutes daily from sedentary time to MVPA was associated with significant improvements in cardiovascular markers, including reductions in LDL cholesterol (−0.18 mmol/L), triglycerides (−0.12 mmol/L), systolic blood pressure (−2.5 mmHg), and increases in HDL cholesterol (+0.10 mmol/L). Substituting sedentary time with light physical activity (LPA) also showed benefits, though to a lesser extent, reinforcing that even small lifestyle changes can have a positive impact [62].

School and community policies encouraging regular physical activity and reducing sedentary behavior are highly relevant for children. In schools, programs incorporating daily physical activities, such as PE classes, active recess, and movement breaks during lessons, can improve children’s cardiovascular health. Creating school environments that limit screen time and promote active play is essential. In the community, the availability of safe, accessible public spaces, such as parks, bike lanes, and sports courts, can encourage physical activity outside school.

Reducing sedentary behavior should also be addressed through educational strategies that raise awareness among families and caregivers about the risks of excessive screen time and the importance of limiting these activities. Awareness campaigns about the benefits of an active lifestyle are necessary and should be incorporated into children’s daily routines, such as promoting active transport (walking and cycling). Integrating these strategies into multisectoral policies can expand their impact, ensuring children have opportunities to move and adopt healthy habits from an early age.

### Health factors and cardiovascular health

Most children in this study had non-HDL cholesterol levels within ideal parameters, while elevated non-HDL cholesterol showed a trend associated with lower prevalence of moderate/high CVH. This finding aligns with previous studies highlighting dyslipidemia as a significant risk factor for cardiovascular disease from childhood that persists into adulthood and increases the risk of early cardiovascular events [63]. Early identification and management of dyslipidemia are essential to reduce the global burden of cardiovascular disease.

Hypertension was present in 30.11% of children. Prevalence of hypertension in children has been documented in various studies, ranging from 4% to 30% depending on the population and diagnostic criteria used [64]. These findings underscore the importance of early blood pressure monitoring, especially in children with risk factors such as obesity and sedentary lifestyle.

### Socioeconomic factors and cardiovascular health

Moderate/high CVH prevalence was higher in children from families with better socioeconomic conditions (AEMC cluster), corroborating previous studies [65,66] highlighting the influence of socioeconomic status on cardiovascular health. This clustering approach was used as an exploratory descriptive summary of socioeconomic profiles and should be interpreted as complementary to conventional socioeconomic indicators. The observed pattern may be explained by several interrelated mechanisms. First, families with higher maternal education and income tend to have greater access to healthy foods, such as fruits, vegetables, and high-quality protein, while lower-income families may rely on cheaper, less nutritious ultraprocessed foods. Maternal education is also associated with greater knowledge of health and nutrition practices, which can positively influence children’s dietary choices.

Another relevant factor is access to safe environments conducive to physical activity. Children from higher-income families often live in neighborhoods with adequate infrastructure, such as parks, bike lanes, and recreational areas, whereas lower-income families may face barriers such as lack of safe spaces or urban violence, limiting opportunities for exercise. Additionally, access to preventive healthcare, such as regular medical consultations and health promotion programs, is more common in families with better socioeconomic conditions, contributing to early detection and management of cardiovascular risk factors.

Policies supporting vulnerable families, such as subsidies for purchasing nutritious foods and expansion of food security programs like the National School Feeding Program (PNAE) [67], can reduce socioeconomic inequalities contributing to childhood obesity. Strengthening the health system, with professional training and expanded access to preventive services, is essential for ensuring comprehensive and effective care.

However, the lack of statistical significance in the multilevel analysis suggests that other factors not captured in this study may influence CVH. For example, cultural aspects, social support networks, and local public policies may play important roles in modulating these associations. Future studies could explore these variables in more detail, as well as investigate how interventions targeting low-income families could reduce disparities in pediatric cardiovascular health.

This study has several strengths worth highlighting. First, the use of a representative sample of Brazilian children, stratified by school type and region, provides a comprehensive view of pediatric cardiovascular health in a context of nutritional transition. Second, applying standardized and validated data collection methods, including anthropometric measurements, blood pressure assessment, and biomarker analysis, ensures result reliability. Third, the inclusion of multiple sociodemographic, behavioral, and biomedical factors in the analysis provides a more holistic understanding of CVH determinants.

The prevalence of overweight children reflects a concerning trend in rising childhood obesity, with direct implications for cardiovascular and public health. Although the association between overweight and low CVH did not reach statistical significance, nearly half of children with overweight and obesity were in this group, compared to 35.35% of children with ideal weight. This may suggest a trend of increased risk for cardiometabolic problems in children with overweight and obesity. Even without statistical significance, children with adequate dietary habits and higher levels of physical activity tend to have better cardiovascular health, reinforcing the need to promote these behaviors from childhood. The results underscore the importance of continuous monitoring of behaviors and health factors in the pediatric population.

Despite these strengths, the study has some limitations. The cross-sectional design prevents causal inference, limiting our ability to establish temporal relationships between DBM and CVH. In particular, the possibility of reverse causality cannot be ruled out, as it is not possible to determine whether DBM influences cardiovascular health or whether pre-existing cardiometabolic alterations may contribute to nutritional imbalances. To minimize this limitation, we adopted a theoretically grounded analytical framework supported by a directed acyclic graph (DAG), which guided covariate selection and reduced inappropriate statistical adjustments.

An additional limitation is the partial overlap between the exposure and outcome definitions in the association analyses. DBM was defined by the coexistence of overweight/obesity and iron deficiency, whereas BMI is also a component of the composite CVH score. This shared component may reduce the independence between exposure and outcome and limits the interpretability of the estimated association, even though no statistically significant association was observed in the main model.

Contextual limitation is that data collection occurred between September 2019 and December 2022, overlapping with the COVID-19 pandemic. Pandemic-related disruptions, including school closures, reduced opportunities for physical activity, changes in daily routines, and increased sedentary behavior, may have influenced behavioral and cardiovascular health indicators in the study population. Therefore, these findings should be interpreted considering the possible impact of this period on children’s lifestyle patterns.

Objective measurements were used for key exposures and outcomes (e.g., accelerometry, biochemical markers, and standardized anthropometry), reducing measurement bias. Nevertheless, longitudinal studies are necessary to clarify temporal directionality and causal pathways. Additionally, other factors not captured in this study, such as diet quality, environmental pollutant exposure, and access to healthcare, may influence CVH more significantly.

## Conclusion

This study showed that most children presented moderate/high CVH; however, factors such as excess weight, sedentary behavior, and inadequate diet may pose risks. The presence of DBM in 4.45% of children highlights the complexity of the nutritional transition in Brazil, emphasizing the coexistence of obesity and nutritional deficiencies. Although no significant association between DBM and CVH was found, this finding underscores the importance of integrated strategies that consider social, behavioral, and biomedical determinants of pediatric cardiovascular health.

The findings reinforce the need for public policies promoting prevention of childhood obesity and adoption of healthy habits from early childhood, including regular physical activity, reduced sedentary time, and improved diet quality. Additionally, interventions that reduce socioeconomic inequalities and expand access to preventive care are crucial for ensuring better cardiovascular health in children. For example, school-based programs that integrate structured daily physical activity, nutrition education focused on reducing ultra-processed food consumption, and routine screening for iron deficiency within primary healthcare services may represent feasible and scalable strategies to address both DBM and cardiovascular risk.

For future directions, longitudinal studies with larger samples are needed to deepen the understanding of CVH determinants in Brazilian children. Further research on DBM is also essential to evaluate its long-term impact and guide effective prevention and management strategies.

## Supporting information

S1 Fig Directed acyclic graph (DAG) representing the assumed relationships among the double burden of malnutrition, cardiovascular health, behavioral factors, and sociodemographic covariates. (PNG)
