# Supplementary figures and images for: Prevalence of the double burden of malnutrition and its association with ideal cardiovascular health in Brazilian children
Source: PLoS One. 2026 Jun 3;21(6):e0349440. doi: 10.1371/journal.pone.0349440 (PMC13232822; doi:10.1371/journal.pone.0349440)

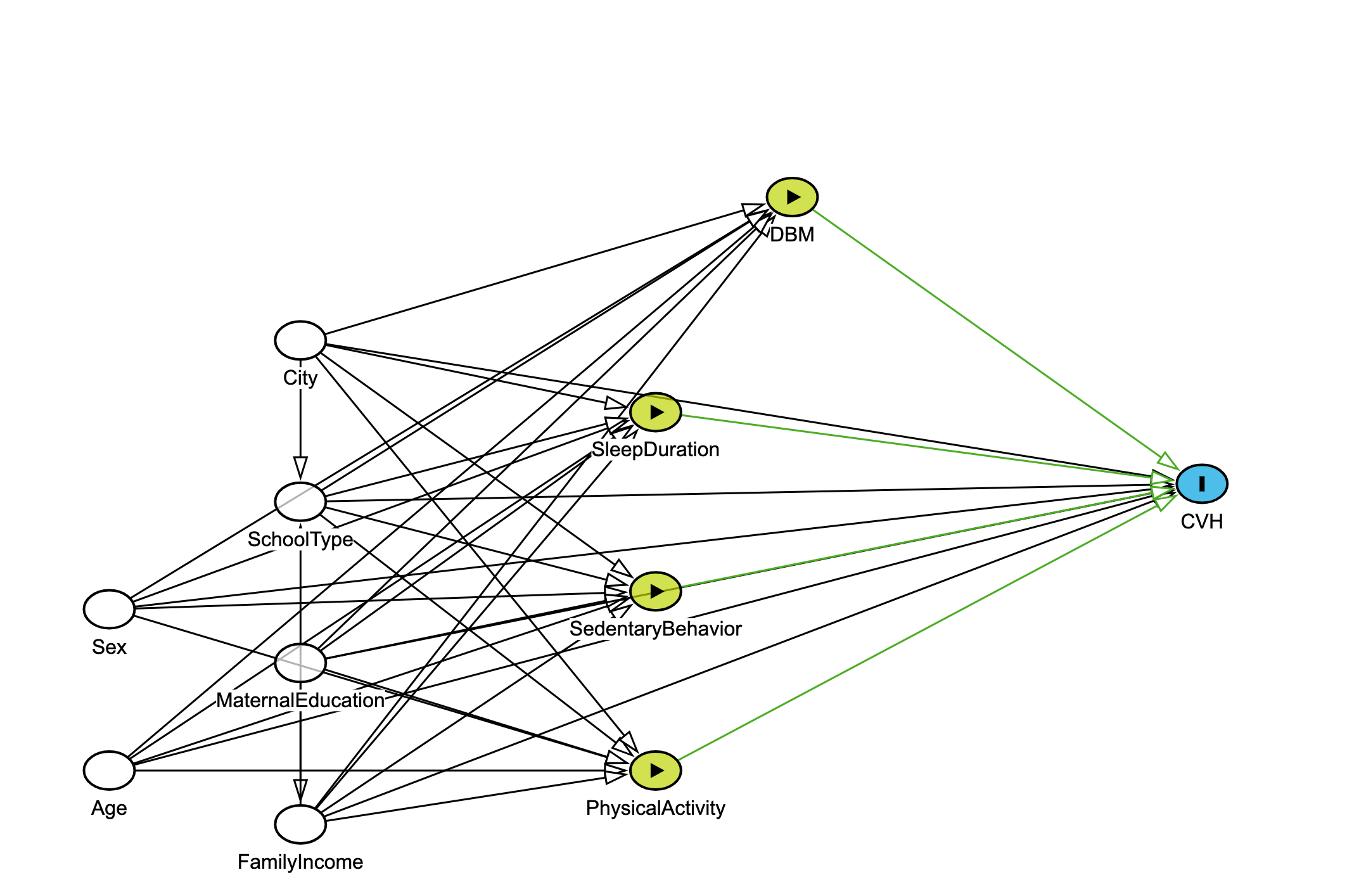

Supplement: S1 Fig — (PNG) [file pone.0349440.s001.png]
